# Progress in Psychiatry

**Published:** 1902-10-25

**Authors:** 


### PROGRESS IN PSYCHIATRY.

(*Concluded from page* 50.)

**The Juvenile Form of General Paralysis.**—Joseph A. Hirschl[6] reports from the clinic of Professor Kraft-Ebing, in Vienna, the details of four cases of juvenile general paralysis, whose ages varied from 16 to 24 years. The most important result of the investigation is shown in the form of a genealogical tree of the patients exhibiting the collateral heredity. In the following case the patient was a boy aged 16 years. At the age of four years he was a well-nourished boy, with slender body and hydrocephalic head. The discs of the eyes were normal. The pupils were unequal, the right being 6 and the left $4\frac{1}{2}$ millimetres in diameter. There was normal reaction to accommodation in the right eye, failing in the left eye. Reaction to light was failing in both eyes. There was tremor of the tongue, speech was bradyphasic and perhaps scanning in quality. There was also present a paralytic disturbance of writing and reading.

Patient's father had cerebral hæmorrhage.
The mother had cataract of the eyes, and was eleven times pregnant.

| | | | | | | | | |
|---|---|---|---|---|---|---|---|---|
| Healthy child, born in 1875. | Ditto, born in 1877. | Abortion at 4 months, in 1878. | Ditto, at 6 months, in 1880. | Patient as described above. Born in 1884. | Full term child died at 4 weeks. Born in 1886. | Ditto, died at 6 months. Born in 1887. | Ditto, died at 3 months. Born in 1888. | Three healthy children, born in 1889, 1891, and 1893, respectively. |

The mother of the above children was syphilitic. In the following case the patient was a boy aged 19 years. His grandfather was epileptic. His father was syphilitic, having contracted the disease in 1868. By his first marriage he begot three children as follows: an abortion in 1872, a still-birth in 1873, and a child born with syphilitic pemphigus in 1874. The pemphigus lasted for about six weeks. The child lived to the age of 26 years, and died of pulmonary phthisis. By his second marriage this man begot six children as follows: the first was a five-months' abortion in 1877, the second a seven-months' still-birth in 1878, the third a similar still-birth in 1879, the fourth was the patient above mentioned (born in 1881), the fifth was a child with syphilitic pemphigus lasting six weeks after birth, born in 1886 and dying at the age of 17 years of phthisis, and the sixth was a child born in 1892 with syphilitic pemphigus, and who died at the age of two years. Dr. Hirschl made twelve autopsies on cases of juvenile general paralysis. He concludes that hereditary syphilis is the chief cause of juvenile general paralysis, that the mental symptoms appear at about puberty, that during childhood the patient shows some weakness of mind, that the prodromal stage is marked by various attacks of partial paralysis, that at its height the disease is a marked and progressive dementia without hypochondriasis or maniacal disturbance as a rule, that the duration of the disease is long and is marked by symptoms of cerebral destruction (such as local paralysis of movement, attacks of mental stupor, or obscuration of consciousness), and that no remissions occur. The autopsies revealed a diffuse lepto-meningitis and encephalitis with morbid adhesions of the pia mater to the cerebral cortex, and the presence of some degree of diffuse cerebral sclerosis.

**The Early Symptoms of General Paralysis.**—In a discussion on general paralysis at the New York Medical Society,[7] Dr. F. X. Dercum, of Philadelphia, dwelt upon the most important diagnostic symptoms of early general paralysis. They were as follows: (*a*) a readily induced fatigue with incapacity for attention, while the patient had a tired and sleepy look; (*b*) attitudes and movements expressive of loss of general muscular tone; (*c*) vague, distressing sensations about the head, such as a feeling of fulness, pressure or constriction of the head, ringing in the ears, attacks of giddiness and vertigo; (*d*) pains in the head, of "lightning-like" or darting character, severe and often simulating migraine; (*e*) the patient rarely comes of his own initiative to the physician, but is brought by friends or relatives; (*f*) the patient is not a hypochondriac or a true neurastheniac; (*g*) the patient's features tend to get flaccid and to lose sharpness of outline; (*h*) the true neurastheniac has simple weakness and readily provoked fatigue, but never the loss of precision and of co-ordination of movements of the general paralytic, and the neurastheniac shows no change in his handwriting or errors in his book-keeping; (*k*) deterioration of his handiwork and manual skill is observed, not by the general paralytic himself, but by his friends or employers; (*l*) the neurastheniac after light and little sleep wakes up "seedy" in the morning, gathers tone and strength as the day wears on and is at his best in the evening, but the general paralytic is, after a variable night of dreams or disturbed sleep, at his best in the morning, loses tone as the day goes on, and may show himself to be stupid, drowsy, thick in speech, and cumbrous in his acts towards evening. The paralytic is also inclined to neglect niceties and proprieties and to exhibit coarseness and indecencies in conduct. Then follow recurring and increasing lapses in conduct and business, and acts of bad taste and lowered morals which are never manifested in neurasthenia. At the recent meeting of the British Medical Association held at Manchester, a discussion on "The Relation of the Functional Neuroses (Hysteria, Neurasthenia, Hypochondriasis, etc.) to Insanity"[8] was opened by Professor Clifford Allbut in which reference was made to the diagnosis of the functional neuroses from early general paralysis of the insane. Early cases of general paralysis might present prodromal, mental and nervous symptoms for some years before definite insanity could be detected. Such prodromal symptoms consisted of incapacity for steady and difficult work, moderate and repeated insomnia, mental exhaustion towards evening, slight attacks of vertigo or unconsciousness, and lapses of memory or of propriety of conduct. M'Corn, of Brooklyn,[9] and Pearce Bailey, of New York[10] both contribute papers on the differentiation of the symptoms of early general paralysis from those of brain syphilis proper. In brain syphilis proper there is intense and boring headache, persistent, and worse at night; while in general paralysis, headache, if present, is usually paroxysmal and localised. Optic neuritis is common in brain syphilis, but rare in general paralysis. The physical symptoms of cerebral syphilis point almost invariably to focal and more or less limited lesions—ptosis, small or partial monoplegias—whereas such focal paralysis do not appear at all in early general paralysis. Brain syphilis also appears within ten years of syphilitic infection, general paralysis after that period. There is also a wide difference as regards the patient's general tone of feeling, apart from any intellectual disturbance. Thus paretics as a rule have a marked euphoria during the onset of the disease, accompanied by exaltation and absurd extravagance of ideas, while the patient with cerebral syphilis is more depressed, and has hypochondriacal, melancholic delusions.

6 Wien. Klin. Woch., No. 21, 1901. 7 Med. News, May 17, 1902. 8 Brit. Med. Jour., August 9, 1902. 9 Brooklyn Med. Jour., February 1902. 10 Med. Rec., June 21, 1902.

